# A Perplexing Case of Recurrent Abscess-Like Lesions: Insights Into Primary Cutaneous Diffuse Large B-Cell Lymphoma, Leg Type

**DOI:** 10.7759/cureus.17766

**Published:** 2021-09-06

**Authors:** Syed Hamza Bin Waqar, Unaiza Zaman, Navid Salahi, Raavi Gupta, Isabel M McFarlane

**Affiliations:** 1 Internal Medicine, State University of New York Downstate Medical Center, New York, USA; 2 Pathology, State University of New York Downstate Medical Center, New York, USA

**Keywords:** pcl, abscess, r-chop, diffuse large b-cell lymphoma, cutaneous lymphoma

## Abstract

Primary cutaneous diffuse large B-cell lymphoma, leg type (PCDLBCL-LT) is one of the rarest forms of primary cutaneous lymphomas (PCLs) and it confers a poor prognosis. Diagnosis of PCDLBCL-LT can be challenging and complex as it can manifest with a myriad of dermatological presentations. However, early treatment with chemo-radiation leads to an appropriate response. We present the case of a 66-year-old female with a history of polymyositis and interstitial lung disease on immunosuppression who presented to our institution with recurrent abscess-like lesions localized to buttocks that were later biopsied and diagnosed as the leg-type variant of PCL. She received chemotherapy with the rituximab plus cyclophosphamide, doxorubicin, vincristine, and prednisone (R-CHOP) regimen and subsequent involved-site radiation therapy (ISRT), which resulted in complete remission. The patient was later followed up and remained in remission for years.

## Introduction

Primary cutaneous lymphoma (PCL) is a group of rare heterogeneous disorders, among which 4% of the cases are contributed by primary cutaneous diffuse large B-cell lymphoma, leg type (PCDLBCL-LT), making it an understudied disease in the literature. PCDLBCL-LT is an aggressive variant that mainly affects the lower extremity but can have multifocal involvement and extracutaneous deposits. In this report, we present a case of a woman with polymyositis and interstitial lung disease who presented to our hospital with a history of recurrent abscess-like lesions and was later found to have PCDLBCL-LT. The patient achieved complete remission with chemo-radiation.

## Case presentation

A 66-year-old female with a known history of interstitial lung disease, polymyositis on immunosuppressants including mycophenolate mofetil, and hypertension presented to our tertiary care hospital with a new abscess on the left buttock. She had experienced recurrent skin abscesses, mostly on the lower abdomen and thigh bilaterally, previously incised and drained for presumed abscess multiple times.

**Figure 1:**
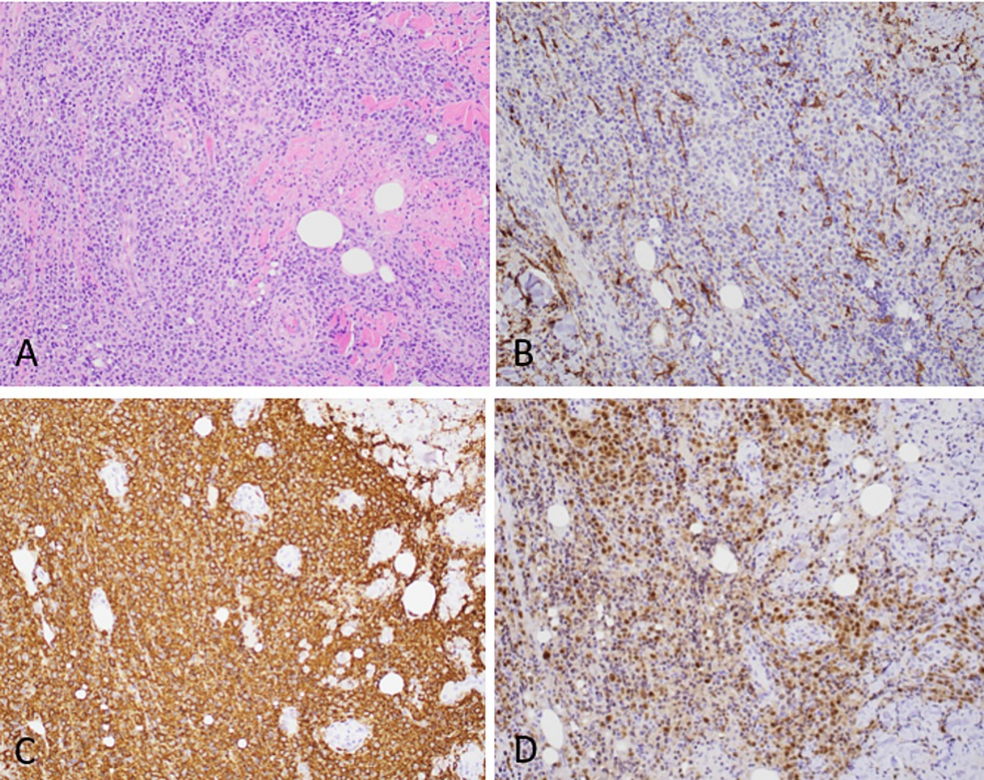
Primary cutaneous diffuse large B-cell lymphoma, leg type (A) This picture shows sheets of centroblasts and immunoblasts in the dermis with necrosis (H&E, 20x). (B) Tumor cells are negative for CD10. (C) Tumor cells are strongly positive for CD20 and (D) MUM1 H&E: hematoxylin and eosin

During her hospitalization, incision, excision, and abscess drainage over the gluteal region were performed and sent for histopathological examination. On immunohistochemistry, the excisional biopsy showed sheets of centroblasts and immunoblasts, positive for CD20 and negative for CD3, CD5, and CD7. These cells stained positive for MUM1, BCL2, and BCL6 (focal) and negative for CD10, consistent with PCDLBCL-LT (Figure 1).

The skin was ulcerated in places with infiltration of lymphoma cells. Since the patient had axillary and inguinal lymphadenopathy, the differential diagnosis included PCDLBCL-LT and the cutaneous manifestation of diffuse large B-cell lymphoma. A positron emission tomography/CT (PET/CT) was performed, which revealed hypermetabolic activity in the left gluteal region [standardized uptake value (SUV) of 6-10] extending to the left gluteal muscle level. Axillary and pelvic lymph nodes were not fluorodeoxyglucose (FDG)-avid. A bone marrow biopsy was performed, which revealed polyclonal plasmacytosis with no evidence of involvement by lymphoma. The patient was staged T1b as the lesion was more than 5 cm in size, with no pathological node involvement or extracutaneous non-lymph node disease.

The patient was started on an anthracycline-based chemotherapeutic regimen [rituximab plus cyclophosphamide, doxorubicin, vincristine, and prednisone (R-CHOP)]. After the first cycle, the patient had a marked decrease in the size of the lesion. She tolerated and completed a total of six cycles of R-CHOP with complete resolution of the cutaneous lesion. The patient also completed adjuvant involved-site radiation therapy (ISRT) and was later followed up at a surveillance clinic with repeat PET/CT scans without relapse for two years.

## Discussion

PCLs are defined as a heterogeneous group of non-Hodgkin lymphomas with cutaneous involvement and no extracutaneous evidence of disease at the time of diagnosis. PCL is broadly characterized into T- and B-cell cutaneous lymphomas, with the latter constituting only 20-25% of all cutaneous lymphomas [1,2]. Both groups of lymphomas have different clinical and pathological characteristics with different treatment protocols and prognoses. These cutaneous lymphomas differ from nodal lymphomas, which may also involve the cutaneous system. Primary cutaneous B-cell lymphoma (PCBCL) has been classified into four main variants: primary cutaneous marginal zone lymphoma (PCMZL), primary cutaneous follicle center lymphoma (PCFCL), PCDLBCL-LT, and Epstein-Barr virus-positive mucocutaneous ulcer (EBVMCU); the latter being included as a provisional entity in the 2016 revision of the World Health Organization-European Organization for Research and Treatment of Cancer (WHO-EORTC) classification [1-3].

PCDLBCL-LT comprises only 4% of all cutaneous lymphomas but portends an aggressive behavior among all the lymphomas, with higher chances of recurrence and relapse. As suggested by the name itself, PCDLBCL-LT mostly affects the lower extremities. However, it can occur at other sites in 15-20% of cases, with specific documented reports suggesting dissemination to extracutaneous sites [4]. It usually presents as erythemato-cyanotic plaques and nodules with rapid growth and needs to be differentiated from somewhat similar PCFCL, which has a much indolent course, different treatment plan, and a somewhat less likely probability of involving extracutaneous sites [2].

Excisional or punch biopsy of cutaneous lesion should be performed, followed by a bone marrow biopsy. Histologically, it comprises diffuse dermal infiltrates of monotonous immunoblasts and centroblasts with high mitotic activity and sparse perivascular T-cell admixture [5]. B-cell lineage markers including CD79a, CD20, and PAX5 are almost always positive, and among germinal center markers, CD10 is absent, and BCL6 is focally present in a handful of cases. FOXP1 and MUM1/IRF4 are also almost always universally present in PCDLBCL-LT [1]. Deletion of CDKN2A at a locus on chromosome 9p21.3 or its promoter hypermethylation is associated with lower disease-specific survival [6]. Other negative prognostic factors include tumor localization on the leg, multifocal tumor, round cell morphology, mutation at MYD88L265P, and high expression of MUM1 and FOXP1. Mutation in MYD88L265P and various components of the B-cell signaling pathway such as CARD11, CD79B, and TNFAIP3/A20 indicate NF-kB activation involvement in leg-type lymphomas [1,5-8].

Treatment for PCDLBCL-LT is still emerging and can be stratified based on solitary regional versus generalized disease based on the literature and evidence. The combination regimen of rituximab with an anthracycline-based combination regimen of CHOP and ISRT is used for both variations, with ISRT used in isolation for patients who might not be eligible for chemotherapy or the solitary variant [9,10]. Therapy with ibrutinib, lenalidomide, bortezomib, and rituximab with PEGylated doxorubicin have also been used with the former three agents for relapsed cases and the last resort for aggressive cases having contraindications to the R-CHOP regimen. Trials with specific monoclonal antibodies like dacetuzumab and lumiliximab are also underway [2,11,12]. Given the belligerent course of illness, surveillance after the chemotherapy should also be aggressive, involving history, physicals, labs, and most importantly, PET/CT imaging.

## Conclusions

PCDLBCL-LT is associated with variability in presentation and can manifest as recurrent abscess-like lesions, and hence must raise concerns for potential malignancy, and excisional biopsies should be sent for histopathological analysis. Timely treatment with the combination of chemotherapy and radiation achieved long-term remission in our case, which adds valuable evidence to the existing body of literature given the scarcity of this type of lymphoma.
